# Study of Endogenous Viruses in the Strawberry Plants

**DOI:** 10.3390/v16081306

**Published:** 2024-08-16

**Authors:** Zongneng Wang, Jian Liu, Xingyang Qi, Daifa Su, Junyu Yang, Xiaolong Cui

**Affiliations:** 1 School of Life Sciences, Yunnan University, Kunming 650500, China; wangzongneng@hos.ynu.edu.cn (Z.W.); liujian_ymq7@stu.ynu.edu.cn (J.L.); liinnian@163.com (X.Q.); dfsu1314@163.com (D.S.); 2 Yunnan International Joint Laboratory of Virology and Immunology, Kunming 650500, China

**Keywords:** plant virus, endogenous viral elements (EVEs), endogenous pararetroviruses (EPRVs), *Caulimoviridae*

## Abstract

Endogenous viral elements (EVEs) have been reported to exist widely in the genomes of eukaryotic organisms, and they are closely associated with the growth, development, genetics, adaptation, and evolution of their hosts. In this study, two methods—homologous sequence search and genome alignment—were used to explore the endogenous viral sequences in the genomes of *Fragaria* species. Results revealed abundant endogenous pararetroviruses (EPRVs) in the genomes of *Fragaria* species, including 786 sequences belonging to five known taxa such as *Caulimovirus* and other unclassified taxa. Differences were observed in the detected EPRVs between the two methods, with the homologous sequence search having a greater number of EPRVs. On the contrary, genome alignment identified various types and sources of virus-like sequences. Furthermore, through genome alignment, a 267-bp sequence with 95% similarity to the gene encoding the aphid-transmitted protein of *Strawberry vein banding virus* (*Caulimovirus venafragariae*) was discovered in the *F. chiloensis* genome, which was likely a recent insertion. In addition, the statistical analysis of the genome alignment results indicated a remarkably higher abundance of virus-like sequences in the genomes of polyploid strawberries compared with diploid ones. Moreover, the differences in virus-like sequences were observed between the genomes of *Fragaria* species and those of their close relatives. This study enriched the diversity of viruses that infect strawberries, and laid a theoretical foundation for further research on the origin of endogenous viruses in the strawberry genome, host–virus interactions, adaptation, evolution, and their functions.

## 1. Introduction

Endogenous viral elements (EVEs) are viral DNA sequences found in the genomes of non-viral organisms, consisting of entire viral genomes or only partial fragments [1,2]. EVEs record past viral infection events, providing “molecular fossils” to study the deep evolutionary history of viruses, and they are an important research tool in the emerging field of “Paleovirology” [3,4]. With the continuous discovery of EVEs, it is known that almost all types of viruses can integrate their nucleic acids into the host genome [5]. A large number of EVEs derived from DNA and RNA viruses are abundant in plant genomes [6,7]. At present, EVEs in plant genomes can be broadly categorized into two groups: those derived from members of the family *Caulimoviridae* are retrotranscribing double-stranded DNA (dsDNA) viruses, which are known as endogenous pararetroviruses (EPRVs), and those that originated from dsRNA, single-stranded RNA (ssRNA), and ssDNA viruses, which are known as endogenous non-retroviral elements (ENREs) [7,8]. The first reported EVE was a geminivirus-related DNA sequence (GRD) [9], whereas EPRVs derived from plant pararetroviruses (PRVs, *Caulimoviridae*) were widely abundant in plant genomes [10].

The Caulimoviridae family consists of 11 genera, including Caulimovirus, Rosadnavirus, Petuvirus, Dioscovirus, Badnavirus, Vaccinivirus, Soymovirus, Tungrovirus, Ruflodivirus, Cavemovirus, and Solendovirus (ICTV) [11]. The nucleotide similarity range of the conserved sequences among the genera of Caulimoviridae has been found to be 42–65%, and the amino acid similarity range 27–48%. In addition, the nucleotide similarity threshold for different species has been found to be 80% [12,13,14]. At present, EPRVs have been found in many plant genomes, such as tobacco [15], Musa spp. [16,17], rice [18,19,20], petunia [21], tomato [22], potato [23], pineapple [24], dahlia [25,26], African yam (Dioscorea cayenensis-rotundata complex) [6], Dracaena sanderiana [27], fig [28], eucalyptus [29], Aristotelia chilensis [30], Citrinae [31], red raspberry [32], alfalfa [8], grapevine [12,33], and eggplant [34]. Large-scale surveys have revealed that EPRVs are widespread in all major plant taxa, including club mosses, ferns, gymnosperms, and angiosperms (including monocotyledon and dicotyledon plants) [34,35,36]. Plant ENREs refer to the internalization of nucleic acids from ssDNA, dsRNA, and ssRNA viruses; plant ENREs primarily match the sequences related to the RNA-dependent RNA polymerase or replicase (Rep), movement protein, and coat protein of the associated viruses [7,37]. Apart from the unclassified ENREs, at least 11 families of ENREs have been reported to be distributed in genomes ranging from algae to higher plants [1,38,39,40,41]. Notably, sequences related to giant virus DNA have also been reported to be integrated into plant genomes, such as large nuclear-cytoplasmic DNA virus-like fragments found in the genomes of Physcomitrella patens and Selaginella moellendorffii [42]. Moniruzzaman et al. (2022) also documented the endogenization of giant viruses in the genomes of Chlamydomonas reinhardtii and Chlamydomonas incerta [40].

Plant EVEs are mainly distributed in plant genomes in the form of fragmentation, and most of them are inactive and only act as “gene fossils”; a small number of EVEs with a complete genome sequence can be assembled into infectious virus particles induced by damage [21], tissue culture [43], species hybridization [15], heat stress [44], sunshine, and seasonal variation [15,45]. To date, three plant EVEs have been activated and have triggered episomal viral infection, including eBSV in the genome of *Musa* spp. [16], eTVCV in the hybrid *Nicotiana edwardsonii* derived from *Nicotiana glutinosa* and *Nicotiana clevelandii* [15], and ePVCV in the hybrid offspring of *Petunia integrifolia* subsp. *inflata* and *P. axillaris* subsp. *axillaris* [21]. The integration of EVEs into or near host genes can be harmful, for example, the insertion of EVEs into the late blight-resistance gene (*R1*) of *Solanum melongena* and its wild relatives has disrupted *R1* orthologs [34]. In addition, 9% of plant endogenous viruses in the grape genome are located in introns, which may affect the expression of host genes [12]. However, most EVEs are neutral and degraded by the accumulation of destructive mutations, insertions, or deletions [10,46]. Based on previous reports, long-term preservation of EVEs in plant genomes can provide plant immunity and resistance to related foreign viruses through homology-dependent transcription or post-transcriptional gene silencing [33]. Plant endogenous viral sequences can also be used as markers to clarify the phylogenetic and evolutionary relationship among plant species, providing unique insights into the evolution and biogeography of viruses and hosts [19,31]. In addition, the integration of endogenous viruses may enlarge the gene pool of plants and endow plants with new functions and stronger adaptation to survival and environment [35,38,47,48,49].

The genus *Fragaria* L. belongs to *Rosaceae*, and it has about 25 species, most of which are wild species. By contrast, *F. × ananassa* is a species that is widely cultivated worldwide [50,51]. Considering asexual reproduction and large-scale greenhouse cultivation, strawberry virus diseases occur frequently, and at least 30 viruses are reported to infect strawberries [52,53]. Among them, *Strawberry vein banding virus* (SVBV, *Caulimovirus venafragariae*), *Strawberry mottle virus* (SMoV, *Sadwavirus fragariae*), *strawberry mild yellow edge virus* (SMYEV, *Potexvirus fragariae*), *Strawberry crinkle virus* (SCV, *Cytorhabdovirus fragariarugosus*), and *Strawberry latent ringspot virus* (SLRSV, *Stralarivirus fragariae*) are the main strains causing strawberry virus diseases worldwide. Viral infections can reduce strawberry fruit yields by 30% to 80% and, in severe cases, by over 80%, posing a threat to strawberry production [54,55]. In addition, Geering et al. (2014) and Tomas et al. (2022) identified endogenous viral sequences integrated into the genomes of *Fragaria* species [10,12]. At present, data on the types, diversity, and classification of endogenous viral sequences in strawberries are still lacking. Thus, in this study, homologous sequence search and genome alignment were used to identify virus-like sequences in the genomes of strawberry plants. The integration differences of virus-like sequences in different strawberry species and their close relatives were compared. Our research expands the diversity of strawberry viruses and deepens the understanding of the integration of endogenous viral sequences and their effects on plants’ genetic evolution.

## 2. Materials and Methods

### 2.1. Materials

The genomes of strawberry and its related genera were obtained first. Considering the genomes of *Duchesnea* species were not found, the genomes of the species from *Potentilla* and *Rubus* were chosen as alternatives. The genomes of 14 *Fragaria* species, two *Potentilla* species, and four *Rubus* species were obtained from the NCBI Genome Database (home-genome-NCBI [nih.gov]) and Rosaceae Genome Database GDR (GDR [rosaceae.org], Supplementary Table S1) [56]. Then, probe sequences, including transcriptase sequences of 11 genera of *Caulimoviridae*, reverse transcriptase (RT) of *Athila4*, and Rep sequences of 14 genera of *Geminiviridae* were obtained from the NCBI protein database (https://www.ncbi.nlm.nih.gov/guide/proteins/ (accessed on 25 May 2023)) and Uniprot protein database (https://www.uniprot.org/ (accessed on 28 June 2023)). In addition, the Rep sequences of non-retroviruses such as SCV, SMoV, and *Fragaria chiloensis cryptic virus* (FCCV) were used to search for homologs (Supplementary Table S2). Furthermore, the NCBI virus genome database viral.1.1.genomic.fna.gz (Index of/refseq/release/viral [nih.gov]) was used for genome alignment.

### 2.2. Viral Homologous Sequence Search

Search, extraction, and clustering of virus-like sequences: First, the Rep sequences of 29 viruses (Supplementary Table S2) were used as the query probe, and the genome of strawberry and its relatives was used as the database for the tBLASTN search. The e-value was set at 1 × 10^−5^. Sequences with at least 50% amino acid similarity and at least 150 amino acid length were selected from the comparison results for further analysis to obtain authentic endogenous viral sequences [12,13,14]. Then, Seq [57] was used to extract viral homologous sequences from each plant genome in batches. Afterward, CD-HIT [58] was used to cluster the extracted sequences with 80% nucleotide similarity to form different clusters.

Phylogenetic analysis: representative sequences from each cluster were selected and added to the prepared RT sequence set (Supplementary Material S1). Then, initial phylogenetic analysis was conducted to eliminate sequences homologous to LTR transposons. The remaining sequences were aligned using MEGA-X software [59], and a maximum likelihood phylogenetic tree was constructed using 500 bootstrap repetitions, with other parameters set to default values. The phylogenetic results served as a classification reference for the extracted endogenous viral sequences. Furthermore, the virus-like sequences in strawberry genomes and the classified EPRVs were counted and analyzed using Excel (Microsoft office 2021).

### 2.3. Alignment of Viral Genomes with the Strawberry Genomes

Using the strawberry genome as a query probe and the viral genome database as the search target, a BLASTN search with the e-value set to 1 × 10^−5^ was conducted. To obtain credible endogenous viral sequences, only sequences that had at least 70% nucleotide similarity to viral genomes and a matching length greater than 100 bp were selected for annotation analysis. Seq was used to extract the selected sequences. Then, phylogenetic analysis was performed on the extracted sequences in the genomes of *Fragaria* species, which are similar to *Caulimoviridae* viruses, as described in Section 2.2. Meanwhile, statistics and an analysis of virus-like sequences in strawberry genomes and EPRVs with the established taxonomic status were performed using Excel (Office 2021).

## 3. Results

### 3.1. Homologous Sequence Search

#### 3.1.1. Integration of Virus-like Sequences in the Genomes of Fragaria and Its Related Genera

In this study, the conserved sequences of viral Reps from 28 genera (six families) (including dsDNA, ssDNA, dsRNA, ssRNA(−), and ssRNA(+) viruses) were used to search for homologs in the genomes of 14 *Fragaria* species, two *Potentilla* species, and four *Rubus* species. The results revealed the presence of *Caulimoviridae* and *Metaviridae* virus-like sequences in the genomes of *Fragaria*, *Potentilla*, and *Rubus*. In particular, a *Fragaria chiloensis cryptic* virus (dsRNA virus) Rep-like sequence was found in the genome of *Potentilla sterilis*, whereas no similar sequences were found in the genomes of *Fragaria* and *Rubus*. Furthermore, the SCV (ssRNA(−) virus) and SMoV (ssRNA(+) virus) Rep-like sequences were not found in the genomes of these three genera of plants (Supplementary Table S3). *Metaviridae* is an important family of LTR retrotransposons, which widely exist in the genome of eukaryotes and play an important part in the eukaryotic genomes. On the contrary, *Caulimoviridae* is a family of plant viruses that can infect plants and cause serious diseases, and it is known as plant pararetrovirus because of its ability to encode RT. Therefore, this study focuses on plant pararetrovirus-like sequences.

#### 3.1.2. Integration and Classification of EPRVs in the Strawberry Genomes

In the strawberry genomes of one cultivated species and 13 wild species, PRV-like sequences were found. Among them, the genomes of *F. nipponica*, *F. pentaphylla*, *F. moupinensis*, and *F. nubicola* contained over 100 sequences, with the *F. moupinensis* genome having 551 sequences. Conversely, the genomes of *F. orientalis* and *F. vesca* had fewer than 50 sequences, with the *F. vesca* genome harboring the lowest count at 37 sequences (Supplementary Table S4). The virus-like sequences in the genomes of each strawberry species were extracted and clustered with an 80% nucleotide similarity threshold, and a representative sequence was selected from each cluster for phylogenetic analysis to remove the sequences that were similar to retrotransposons, thereby obtaining the EPRV sequences, that is, the effective sequences in this study. A total of 779 EPRV sequences were obtained from strawberry genomes and clustered into 79 clusters. Similarly, the number of EPRVs in the genomes of *F. nipponica*, *F. pentaphylla*, *F. moupinensis*, and *F. nubicola* exceeded 50, with the *F. moupinensis* genome containing up to 276 sequences. Consistent with the virus-like sequences, the *F. vesca* genome harbored the fewest endogenous plant pararetrovirus sequences at 16 (Supplementary Table S4).

**Figure 1:**
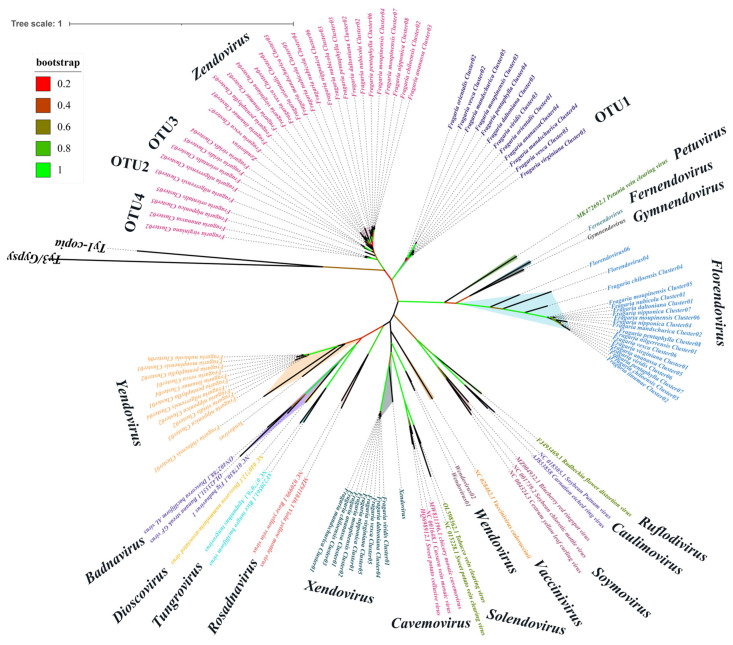
The phylogenetic relationship of strawberry pararetroviral RT homologs within the *Caulimoviridae* family, as retrieved from a homology search. The maximum likelihood tree was constructed using representative sequences derived from strawberry genomes and 11 genera of exogenous viral species within the *Caulimoviridae* family (per ICTV classification), along with RT sequences from 7 endogenous viral genera. The phylogenetic analysis was bootstrapped 500 times, and the confidence level of the branch was indicated by the color of the branch. The 11 exogenous viral genera (ICTV) include: *Caulimovirus*, *Rosadnavirus*, *Petuvirus, Dioscovirus*, *Badnavirus*, *Vaccinivirus*, *Soymovirus*, *Tungrovirus*, *Ruflodivirus*, *Cavemovirus*, and *Solendovirus*. The 7 endogenous viral genera comprise: *Florendovirus*, *Gymnendovirus*, *Fernendovirus*, *Yendovirus*, *Zendovirus*, *Xendovirus*, and *Wendovirus.* The Ty1-copia and Ty3/Gypsy retrotransposon were selected as outgroups, and the rooted tree constructed by phylogenetic analysis was shown in Supplementary Figure S1.

A representative sequence was selected from each cluster for phylogenetic analysis to determine the taxonomic position of EPRVs in the strawberry genomes (Figure 1 and Supplementary Figure S1). Based on the phylogenetic analysis results, the 779 EPRV sequences identified in the strawberry genomes were categorized into eight taxonomic groups, including four known endogenous plant pararetrovirus groups (*Yendovirus*, *Zendovirus*, *Florendovirus*, and *Xendovirus*) and four unknown groups labeled as VOTU1-OTU4 (Supplementary Figure S1). The phylogenetic tree revealed that VOTU1 branched independently, indicating a new taxonomic group. Meanwhile, VOTU2-OTU4 was positioned on the same evolutionary branch as the *Zendovirus* group, indicating a close relationship between them (Figure 1). Notably, the RT homologs of these plant pararetroviruses did not belong to any known taxonomic group within *Caulimoviridae* (per ICTV classification).

#### 3.1.3. Diversity and Differences of EPRVs in Strawberry Genomes

**Figure 2:**
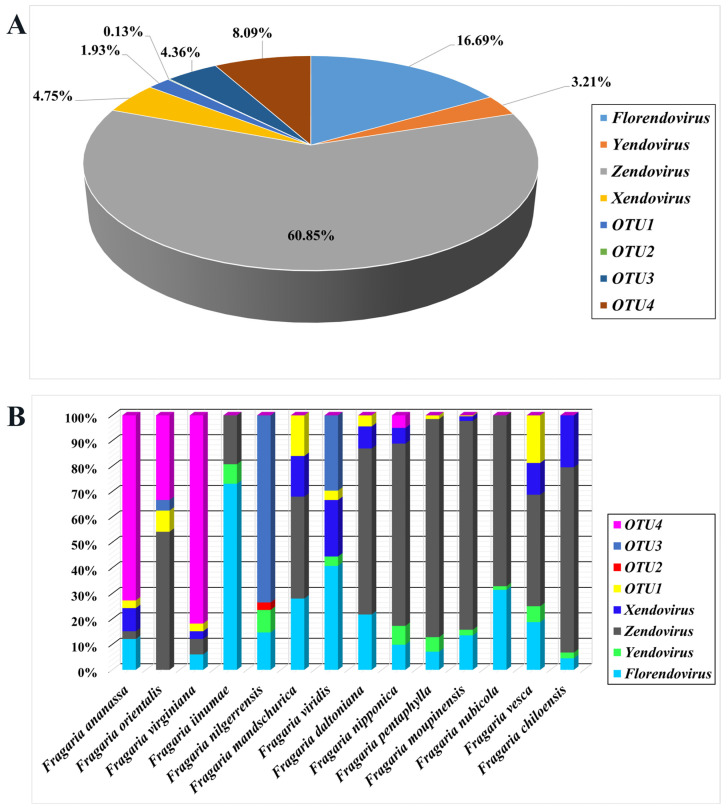
Classification statistics of strawberry EPRVs obtained by homologous search (**A**); composition of EPRVs in different strawberry genomes (**B**).

The EPRV sequences in strawberry genomes were statistically analyzed. The results indicated that the most abundant EPRVs belonged to *Zendovirus*, accounting for 60.85%, followed by *Florendovirus*, accounting for 16.69%. The smallest group was VOTU2, accounting for 0.13%, followed by VOTU1, accounting for 1.93% (Figure 2A). From different strawberry species, *Zendovirus* and *Florendovirus* are integrated into the genomes of almost all strawberry plants. Zendovirus is the main group integrated into the genomes of *F. orientalis*, *F. mandschurica*, *F. daltoniana*, *F. nipponica*, *F. pentaphylla*, *F. moupinensis*, *F. nubicola*, *F. vesca*, and *F. chiloensis*. *Florendovirus* is the main group integrated into the genomes of *F. iinumae*. Furthermore, the unclassified taxonomic group VOTU4 was identified as a predominant clade in the genomes of *F. virginiana* and cultivated strawberry *F. × ananassa* Duch. cv. Wongyo 3115 [60], while being present in the genomes of *F. nipponica* and *F. orientalis*. The unknown taxon VOTU3 is the main group integrated into the genome of *F. nilgerrensis*, and this group is also integrated into the genomes of *F. viridis* and *F. orientalis*. Notably, the unknown taxa VOTU2 is only found in the genome of *F. nilgerrensis* (Figure 2B).

### 3.2. Genome Alignment Results

#### 3.2.1. Virus-like Sequences and Differences in the Genomes of Strawberries

**Figure 3:**
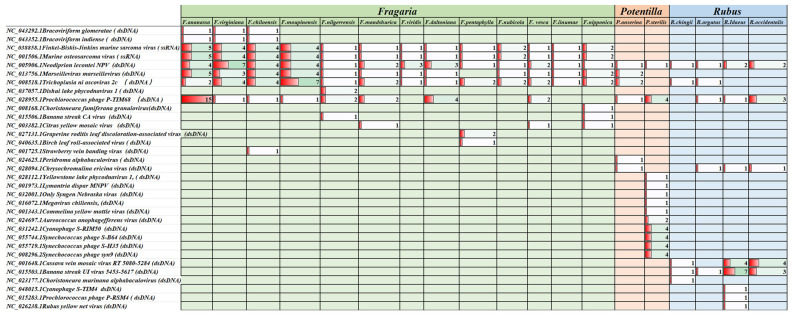
Statistical diagram of the genome alignment results. Only sequences with at least 70% nucleotide similarity to the viral genome and at least 100 nucleotides in length were counted. Because the *F. orientalis* genome was only assembled to the scaffold level, to avoid errors the alignment results of its genome were not counted when comparing the differences in similar viral sequences between different species.

Genome alignment directly aligned the genomes of strawberries and other related plants to the NCBI viral genome database to identify virus-like sequences. In this study, the genomes of 14 *Fragaria* species, two *Potentilla* species, and four *Rubus* species were aligned with the NCBI viral genome database viral. 1.1. genomic.fna.gz (Index of/refseq/release/viral [nih.gov]), and sequences with at least 100 bp length and 70% similarity were considered as potential viral integration sequences. The results indicate that 15 different viral genome segments were found in 13 strawberry genomes (Figure 3). Among them, five virus-like fragments could be found in almost all strawberry plant genomes, including *Finkel-Biskis-Jinkins murine sarcoma virus*, *Murine osteosarcoma virus*, *Neodiprion lecontei nucleopolyhedrovirus*, and *Trichoplusia ni ascovirus 2c*. *Prochlorococcus phage P-TIM 68*-like sequences were also found in the genomes of eight strawberry species. Sequences similar to *Cotesia glomerata bracovirus* and *Glyptapanteles indiensis bracovirus* were found in the genomes of *F. ananassa* Duch. cv. Wongyo 3115, *F. chiloensis*, and *F. virginiana*. Sequences similar to *Banana streak CA virus* were present in the genome of *F. nilgerrensis* and *F. nipponica*. Sequences similar to *Citrus yellow mosaic virus* were found in the genomes of *F. orientalis*, *F. nipponica*, and *F. vesca*. In addition, some viral genome sequences could only be found in a specific strawberry genome, for example, *Choristoneura fumiferana granulovirus* only had similar fragments in the *F. nipponica* genome *Dishui lake phycodnavirus 1*. Similar sequences were only found in the *F. nilgerrensis* genome. *Grapevine roditis leaf discoloration-associated virus* (*Caulimoviridae*) only had similar fragments in the *F. pentaphylla* genome, and *Birch leaf roll-associated virus* (*Caulimoviridae*) only had similar fragments in the *F. pentaphylla* genome. This study also found fragments similar to the SVBV genome in the *F. chiloensis* genome, with 95% similarity and 267 bp alignment length. Notably, the complete genome of *Escherichia phage phiX174* was assembled from the genome of *F. orientalis* (Supplementary Material S2 and Supplementary Figure S2).

#### 3.2.2. Comparison of Virus-like Sequences in Different Plant Genomes

The differences and similarities in virus-like sequences among plant genomes of three genera are evident, with distinct yet shared similarities. Statistical analysis revealed variations in the virus-like sequences aligned in the genomes of strawberries and their relatives. Concurrently, some similar virus-like sequences were matched in the genomes of strawberries and their close relatives, including *Neodiprion lecontei NPV*, *Trichoplusia ni ascovirus 2c*, and *Prochlorococcus phage P-TIM68*. With regard to viral nucleic acid types, the virus-like sequences identified in the genomes of the three genera predominantly belonged to dsDNA viruses, with a minority attributed to ssRNA viruses (Figure 3).

**Figure 4:**
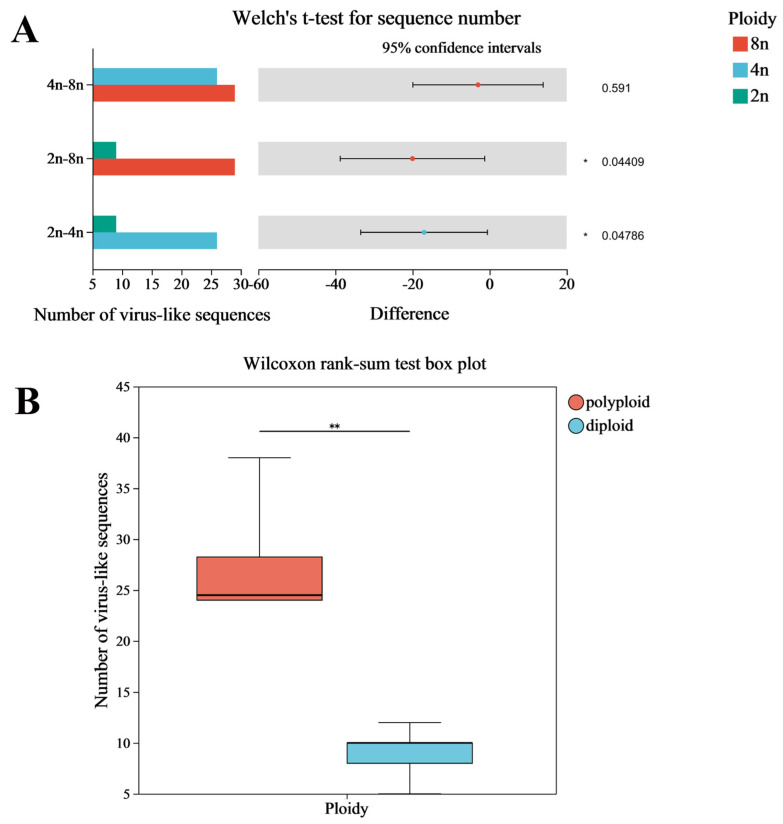
Welch’s *t*-test of endogenous virus-like sequences in different ploidy strawberry genomes (**A**); Wilcoxon rank-sum test analysis of endogenous virus-like sequences in the genomes of polyploid (octaploid and tetraploid) and lowploid (diploid) strawberries (**B**). The magnitude of the significance *p*-value was indicated by “*” (“*”, 0.01 < *p* < 0.05; “**”, 0.001 < *p* < 0.01).

Furthermore, differences in the quantity of virus-like segments were found in the genomes of different strawberry species, exhibiting a correlation with the ploidy level of the strawberry species. In particular, the number of virus-like segments in high-ploidy strawberry genomes was higher than that in low-ploidy strawberries. However, no variation in the quantity of virus-like sequences was found in high-ploidy strawberry genomes. An analysis of virus-like sequences in genomes of strawberries with different ploidy levels revealed that the number of virus-like segments in the octoploid strawberries *F. virginiana* and *F. chiloensis*, the cultivated *F. × ananassa* Duch. cv. Wongyo 3115 from Japan, and the tetraploid strawberry *F. moupinensis* was higher than that in the remaining diploid strawberries. The results from conducting Welch’s *t*-test and the Wilcoxon rank-sum test analysis to compare the differences in the number of virus-like segments among genomes of strawberries with different ploidy levels indicated significant differences in the number of virus-like sequences between tetraploid and diploid strawberry genomes, as well as between octoploid and diploid strawberry genomes. However, no significant difference was observed in the number of virus-like sequences between tetraploid and octoploid strawberry genomes (Figure 4A). Overall, the number of virus-like segments in the highly ploid strawberry genomes was significantly higher than that of the ordinary diploid strawberry (Figure 4B).

#### 3.2.3. Integration and Classification of EPRVs in the Strawbery Genomes (Identification of EPRVs in the Strawberry Genomes)

Based on the species annotation results, segments similar to pararetroviruses in plant genomes were further analyzed, and the functional regions of these similar segments in the virus genome were determined through alignment. The results revealed that the region 22,846,325–22,846,439 on chromosome 4 of *F. nipponica* and the region 35,020,921–35,021,035 on chromosome 3 of *F. nilgerrensis* showed 80% and 81% alignment similarity, respectively, to regions 5570–5684 in the *Banana streak CA virus* genome. Upon inspecting the virus genome maps, these regions were found to correspond to the RT sequence of *Banana streak CA virus*. Similarly, the regions 18,447,245–18,447,362 on chromosome 5 of *F. orientalis*, 10,881,625–10,881,742 on chromosome 1 of *F. nipponica*, and region 536,392–536,509 on the *F. vesca* genome sequence GG775297.1 exhibited 79%, 80%, and 79% alignment similarities, respectively, to the RT region 5586–5703 of *Citrus yellow mosaic virus*. Furthermore, the region 29,284,759–29,284,886 on chromosome 2 of *F. pentaphylla* showed 78% alignment similarity to the RT of *Grapevine roditis leaf discoloration-associated virus*, and the region 16,387,420–16,387,565 on chromosome 4 showed 77% alignment similarity to the RT of *Birch leaf roll-associated virus*. In addition, the region 20,067,530–20,067,795 on chromosome Fchil2-B2 of *F. chiloensis* exhibited 95% alignment similarity to the aphid-transmission-associated protein sequence of SVBV (Table 1).

**Figure 5:**
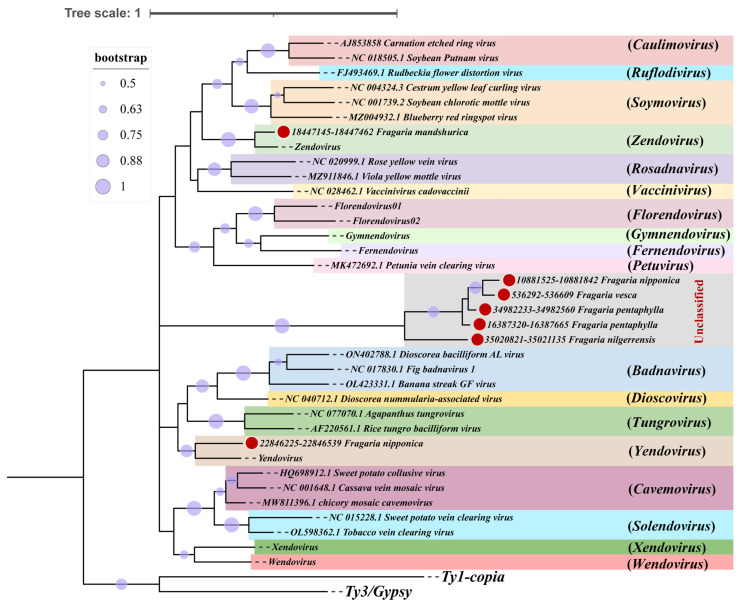
Phylogenetic analysis of strawberry endogenous pararetrovirus sequences obtained from genomic alignments. The root tree was constructed using the maximum likelihood method by MEGA-X software. The retrotransposons Ty 1-copia and Ty 3/Gypsy were selected as outgroups. The pararetrovirus sequences extracted from the strawberry genomes are marked with red dots. Bootstrap is indicated by the circle size.

Fragments of plant pararetroviruses in the strawberry genomes were extracted, with 100 bp extensions upstream and downstream, for phylogenetic analysis using MEGAx. Phylogenetic analysis revealed that plant pararetroviruses, such as sequences on chromosome 5 of *F. orientalis*, belonged to the classification group of *Zendovirus*. The sequences on chromosome 4 of *F. nipponica* were classified under *Yendovirus*. The plant pararetroviruses, such as sequences on chromosome 3 of *F. nilgerrensis*, chromosome 1 of *F. nipponica*, chromosome 2 and chromosome 4 of *F. pentaphylla*, and chromosome GG775297.1 of *F. vesca* were categorized as an unknown classification group (Figure 5).

**Figure 6:**
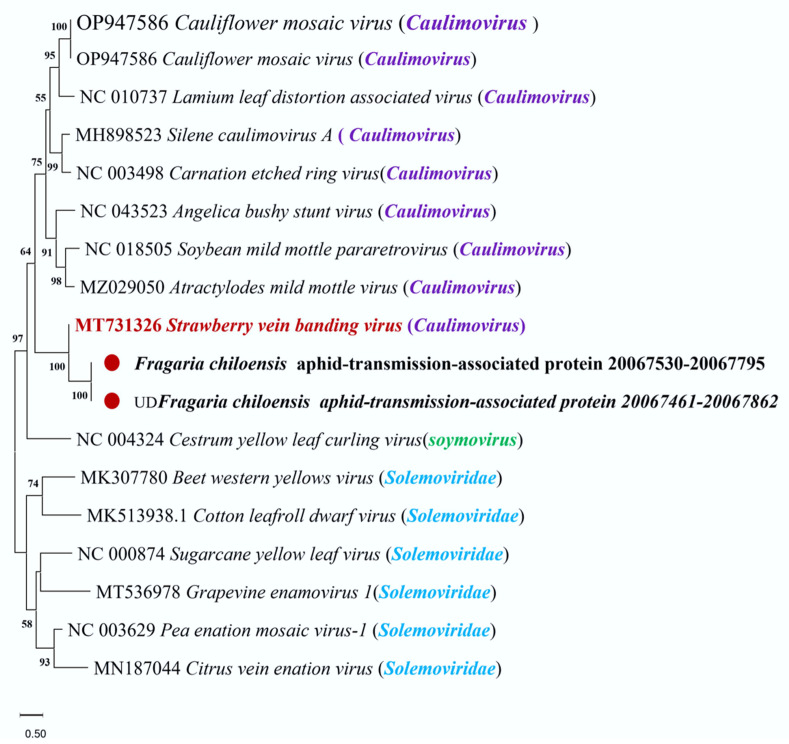
Phylogenetic analysis of aphid transmission-related protein gene sequences extracted from the *F. chiloensis* genome. The root tree was constructed using the maximum likelihood method by MEGA-X software. Virus-like sequences extracted from the *F. chiloensis* genome are marked with red dots. The sequences of the remaining genes encoding aphid-transmitted proteins were obtained from the NCBI database. “UD” represents the sequence resulting from extending the alignment hit with appropriate lengths upstream and downstream.

A segment of the aphid-transmission-associated protein sequence from SVBV was discovered in the *F. chiloensis* genome. SVBV belongs to the *Caulimovirus* genus of the *Caulimoviridae* family, it infects strawberries, and it can be transmitted by aphids. In this study, the region 20,067,530–20,067,795 on chromosome Fchil2-B2 of *F. chiloensis* shows 95% alignment similarity to the region 1133–1398 of the aphid-transmission-associated protein gene in the genome of SVBV. A phylogenetic analysis was conducted using the aphid-transmission-associated protein sequence belonging to *Caulimoviridae* viruses. The phylogenetic analysis results indicate that the aphid-transmission-associated protein-like sequence found in the *F. chiloensis* genome clustered with the aphid-transmission-associated protein sequence of SVBV, indicating that the similar sequence in the *F. chiloensis* genome represents an integrated sequence of the aphid-transmission-associated protein related to SVBV (Figure 6).

### 3.3. Comparison of the Two Study Methods

After searching for homologs of viral Reps (sequences with at least 50% amino acid similarity and at least 150 amino acid length were selected), homologs of plant pararetrovirus (*Caulimoviridae*) RT were identified in the strawberry genomes. These RT homologs found in the genomes of 14 species of *Fragaria* belong to eight taxonomic groups, totaling 779 sequences. These groups include *Yendovirus*, *Zendovirus*, *Florendovirus*, *Xendovirus*, and four unclassified groups (VOTU1-OTU4). By directly aligning the strawberry genomes with viral genome databases (sequences with at least 70% nucleotide similarity and at least 100 nucleotides in length were selected), plant pararetrovirus-like and non-retroviral virus-like sequences were identified in the strawberry genomes. Among these, the identified plant pararetroviruses were obtained from *Yendovirus*, *Zendovirus*, *Caulimovirus*, and an unclassified taxon, totaling eight sequences. These sequences were derived from the RT homologs of plant pararetroviruses (*Caulimoviridae*) and the aphid-transmitted protein homologs of *Caulimovirus*. In addition, the complete genome of *Escherichia phage phiX174* was assembled from the genome of *F. orientalis* by genome alignment. Overall, the search results differ between the two methods. Homolog searches are more specific, whereas genome alignment is more comprehensive. Therefore, combining multiple methods is necessary for studying plant EVEs.

## 4. Discussion

Replicase is a conserved sequence of viruses, which is often used in virus phylogenetic analysis and homology searches [36,61]. Rep sequences of *Caulimoviridae*, *Geminiviridae*, and other non-retroviruses were used to search for homologs in the genome of *Fragaria* species. The results indicated the presence of RT homologs of *Caulimoviridae* viruses in the genomes of strawberry plants. The number of RT homologs in the genomes of different strawberry species varied from 16 to 276, whereas several EPRV fragments were reported from other plants [31,36]. Based on our results, no homologs of *Geminiviridae* virus or other non-retroviral virus Reps were found in the genomes of *Fragaria* species, despite their presence in different plant species [39,61,62,63]. Plant pararetroviruses differ from animal retroviruses in that integrating their genomes into the host genome is not essential for their life cycle [10,64]. In addition, most EVEs are discontinuous, rearranged viral gene fragments [12]. Therefore, reconstructing complete viral genomes of plant viruses through homologous sequence searches is challenging [61,65]. Thus, we aligned the strawberry plant genomes directly with the viral genome database to match and assemble the complete viral genome. Following our selection criteria, where sequences below 70% nucleotide similarity and shorter than 100 bp were discarded, a complete genome belonging to the *Caulimoviridae* virus in the genomes of *Fragaria* species was not obtained. However, Geering et al. (2014) successfully assembled a complete viral genome in the *F. vesca* genome [12]. This discrepancy may be attributed to our selected thresholds and the selection of effective sequences. We also managed to assemble the complete genome of *Escherichia phage phiX174* [66,67] in the genome of *F. orientalis* (Supplementary Material S2 and Supplementary Figure S2). As the genome of *F. orientalis* has only been assembled to the scaffold level [68], this occurrence may have resulted from errors during sequencing and genome assembly, which warrants further validation through subsequent research.

In this study, the differences in virus-like sequences were compared between the genomes of *Fragaria* species and closely related genera (*Potentilla* and *Rubus*). The results indicated differences in virus-like sequences among the genomes of *Fragaria*, *Potentilla*, and *Rubus* species. In addition, some common virus-like sequences were shared between the genomes of *Fragaria* species and those of *Potentilla* and *Rubus*. These virus-like sequences could be ancient viral sequences that infected and integrated into their common ancestral genome [19]. The viral sequences integrated into the host genome can serve as markers to study the phylogeny, adaptation, and evolutionary relationships among plant species, as well as infer the activity of ancient viruses, their geographical origins, and integration history [19,31]. Therefore, the identification of virus-like sequences in the genomes of *Fragaria* and closely related species could be highly valuable for conducting research on the genetics, adaptation, evolution, phylogeny, and speciation of strawberry species. Furthermore, by aligning the genomes of *Fragaria* species directly with viral genome databases, the number of virus-like segments in the genomes of polyploid strawberries (such as tetraploid strawberries [*F. orientalis* and *F. nilgerrensis*] and octoploid strawberries [*F. × ananassa*, *F. virginiana*, and *F. chiloensis*]) was found to be significantly higher than that in diploid strawberries (such as *F. nilgerrensis* and *F. pentaphylla*). At present, the endogenization of viral nucleic acids in plant genomes was due to non-homologous recombination between virus and host genomes, which often occurs during double-strand DNA break repair or transposon-mediated recombination [6,61]. Therefore, polyploids with a number of chromosomes increased the probability of viral nucleic acid insertion, leading to the higher abundance of virus-like segments in polyploid strawberries compared with diploids.

SVBV is a plant pararetrovirus belonging to the *Caulimovirus* genus that can be transmitted by aphids to infect strawberries [69]. The aphid transmission factor of SVBV plays an important role in its infection and transmission processes [70,71]. Through genome alignment, we identified a homologous sequence of the aphid-transmission-associated protein of SVBV integrated into the genome of *F. chiloensis*. This segment is 267 bp long and shares 95% similarity with the aphid transmission protein sequence of SVBV, suggesting a recent insertion. We hypothesized that the integration of the homolog of the aphid-transmission-associated protein of SVBV may confer immune resistance to SVBV in *F. chiloensis*, although the transcriptional activity of this segment needs further verification [14,22,72].

In our report, two methods were used to search for virus-like sequences in the genomes of *Fragaria* species. In identifying highly homologous EPRVs, sequences with a minimum of 50% amino acid similarity and at least 70% nucleotide similarity were retained as EPRV-like sequences [12,13]. Using this threshold, our homology searches in the strawberry genome identified EPRVs belonging to *Yendovirus*, *Zendovirus*, *Florendovirus*, *Xendovirus*, and four unknown taxonomic groups VOTU1–OTU4 (with VOTU1 being a potential novel group). Compared with our results, *Tungrovirus* has been reported to be integrated into strawberry genomes [10]. In contrast to homology searches, genome alignment revealed the presence of *Caulimovirus*, *Yendovirus*, *Zendovirus*, and an unknown taxonomic group. Our results have expanded the diversity of viruses in the genomes of *Fragaria* species. Our research also suggested that different approaches to searching endogenous viral sequences in plant genomes yield distinct results, emphasizing the importance of combining multiple methods for an accurate and comprehensive characterization of EVEs in plant genomes.

## 5. Conclusions

In this study, a homologous sequence search and genome alignment were used to study the endogenous viral sequences in strawberry genomes. A notable diversity of EPRVs and potential new taxa of *Caulimoviridae* were identified in strawberry genomes. By comparing virus-like sequences in the genomes of *Fragaria* species and those of closely related plants, variations in the composition of virus-like sequences were found among different plant genera, while shared virus-like sequences were observed among closely related plant genera. Furthermore, we reported a nucleotide sequence from the aphid-transmission-associated protein of *Strawberry vein banding virus* in the genome of *F. chiloensis*, which may be related to the resistance of *F. chiloensis* to *Strawberry vein banding virus*. At present, the impact of the integration of these viral elements on strawberries needs further study.

## Figures and Tables

**Table 1:** Statistical analysis of plant pararetrovirus-like sequences identified by genome alignment in the strawberry genomes.

Position in the Strawberry Genomes	Position in the Virus Genomes	Similarity	Length	Functional Domain	Virus Genome Accession No
*Fragaria mandshurica* chr5 18,447,245–18,447,362	*Citrus yellow mosaic virus* 5586–5703	79%	119 bp	RT	NC_003382.1
*Fragaria nipponica* chr1 10,881,625–10,881,742	*Citrus yellow mosaic virus* 5586–5703	81%	119 bp	RT	NC_003382.1
*Fragaria vesca* GG775297.1 536,392–536,509	*Citrus yellow mosaic virus* 5586–5703	79%	119 bp	RT	NC_003382.1
*Fragaria nipponica* chr4 22,846,325–22,846,439	*Banana streak CA virus* 5570–5684	80%	119 bp	RT	NC_015506.1
*Fragaria nilgerrensis* chr3 35,020,921–35,021,035	*Banana streak CA virus* 5570–5684	81%	119 bp	RT	NC_015506.1
*Fragaria pentaphylla* chr2 29,284,759–29,284,886	*Grapevine roditis leaf discoloration-associated virus* 5180–5307	78%	133 bp	RT	NC_027131.1
*Fragaria pentaphylla* chr4 16,387,420–16,387,565	*Birch leaf roll-associated virus* 5947–6092	77%	149 bp	RT	NC_040635.1
*Fragaria chiloensis* Fchil2-B2 20,067,530–20,067,795	*Strawberry vein banding virus* 1133–1398	95%	267 bp	Aphid transmission associated protein	NC_001725.1

## Data Availability

The data presented in this study are available in NCBI Genome Database(home-genome-NCBI [nih.gov]), reference number [GCA_019022445.1, GCA_000517285.1, GCA_009720345.1, GCA_010134655.1, GCF_000184155.1, GCA_933775445.1, GCA_963682095.1]; NCBI Protein Database (https://www.ncbi.nlm.nih.gov/guide/proteins/ (accessed on 25th May 2023)), reference number [NP_056728.1, YP_007761644.1, Q6XKE6, YP_009553219, AF378081.1, AAP03645.2, QPZ44443.1, QZN83648.1]; Uniprot protein database (https://www.uniprot.org/ (accessed on 28 June 2023)), reference number [A0A2H4N978, A0A0S2A4A7, G5CKJ8, C4QUK2, B8Y871, E5KBV1, Q9QD04, A0A166V1S2, A0A858M4E9, P0CK40, B1P3D6, P14991, C3UV60, T2B2N5, P14988, A0A6C0M7U4, A0A2R4Q8U9, A0A3S9JKY8, Q88888, A0A0S3JNW4]; and Rosaceae Genome Database GDR (GDR [rosaceae.org].

## Supplementary Materials

The following supporting information can be downloaded at: https://www.mdpi.com/article/10.3390/v16081306/s1. Supplementary Material S1: RT sequence set for phylogenetic analysis, Supplementary Material S2: Genome sequence of *Escherichia phage phiX174* assembled from the genome of *Fragaria orientalis*, Supplementary Figure S1: the rooted tree of strawberry pararetroviral RT homologs within the *Caulimoviridae* family, got from homology search, Supplementary Figure S2: Genomic illustration of *Escherichia phage phiX174* assembled from the genome of *Fragaria orientalis*, Supplementary Table S1: The plant genomes of *Fragaria* and its related genera used in this study, Supplementary Table S2: The probe sequences used for homologous search, Supplementary Table S3: Overview of the search for homologs of viral replicases in the genomes of the *Fragaria* and its closely related plant genera, Supplementary Table S4: Statistical results of plant pararetrovirus-like sequences and effective sequences identified by homologs search in the strawberry genomes.
