# Supplementary material for: Study of Endogenous Viruses in the Strawberry Plants
Source: Viruses. 2024 Aug 16;16(8):1306. doi: 10.3390/v16081306 (PMC11359110; doi:10.3390/v16081306)
Supplement: Supplementary file 1 [file viruses-16-01306-s001.zip › Supplementary Figure s2.pdf]

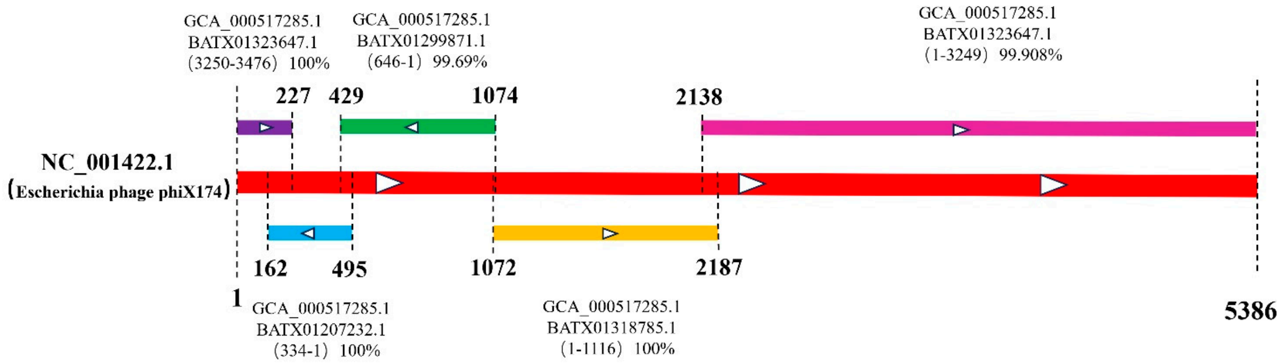

supplementary Figure s2: Genomic illustration of *Escherichia phage phiX174* assembled from the genome of *Fragaria orientalis*.
