# Supplementary material for: Study of Endogenous Viruses in the Strawberry Plants
Source: Viruses. 2024 Aug 16;16(8):1306. doi: 10.3390/v16081306 (PMC11359110; doi:10.3390/v16081306)
Supplement: Supplementary file 1 [file viruses-16-01306-s001.zip › Supplementary material s2_Genome sequence of Escherichia phage phiX174 assembled from the genome of Fragaria orientalis/Genome sequence of E.pdf]

整合病毒完整基因组

GAGTTTTATCGCTTCCATGACGCAGAAGTTAACACTTTCGGATATTTCTGATGAGTCGAAAAATT
ATCTTGATAAAGCAGGAATTACTACTGCTTGTTTACGAATTAAATCGAAGTGGACTGCTGGCGG
AAAATGAGAAAATTCGACCTATCCTTGCGCAGCTCGAGAAGCTCTTACTTTGCGACCTTTCGCCA
TCAACTAACGATTCTGTCAAAAACTGACGCGTTGGATGAGGAGAAGTGGCTTAATATGCTTGGC
ACGTTCGTCAAGGACTGGTTTAGATATGAGTCACATTTTGTTCATGGTAGAGATTCTCTTGTTGAC
ATTTTAAAAGAGCGTGGATTACTATCTGAGTCCGATGCTGTTCAACCACTAATAGGTAAGAAATC
ATGAGTCAAGTTACTGAACAATCCGTACGTTTCCAGACCGCTTTGGCCTCTATTAAGCTCATTCA
GGCTTCTGCCGTTTTGGATTTAACCGAAGATGATTTCGATTTTCTGACGAGTAACAAAGTTTGGA
TTGCTACTGACCGCTCTCGTGCTCGTCGCTGCGTTGAGGCTTGCGTTTATGGTACGCTGGACTTT
GTAGGATACCCTCGCTTTCCTGCTCCTGTTGAGTTTATTGCTGCCGTCATTGCTTATTATGTTCAT
CCCGTCAACATTCAAACGGCCTGTCTCATCATGGAAGGCGCTGAATTTACGGAAAACATTATTA
ATGGCGTCGAGCGTCCGGTTAAAGCCGCTGAATTGTTCGCGTTTACCTTGCGTGTACGCGCAGG
AAACACTGACGTTCTTACTGACGCAGAAGAAAACGTGCGTCAAAAATTACGTGCAGAAGGAGT
GATGTAATGTCTAAAGGTAAAAAACGTTCTGGCGCTCGCCCTGGTCGTCCGCAGCCGTTGCGAG
GTACTAAAGGCAAGCGTAAAGGCGCTCGTCTTTGGTATGTAGGTGGTCAACAATTTTAATTGCA
GGGGCTTCGGCCCCTTACTTGAGGATAAATTATGTCTAATATTCAAACTGGCGCCGAGCGTATG
CCGCATGACCTTTCCCATCTTGGCTTCCTTGCTGGTCAGATTGGTCGTCTTATTACCATTTCAACT
ACTCCGGTTATCGCTGGCGACTCCTTCGAGATGGACGCCGTTGGCGCTCTCCGTCTTTCTCCATT
GCGTCGTGGCCTTGCTATTGACTCTACTGTAGACATTTTTACTTTTTATGTCCCTCATCGTCACGTT
TATGGTGAACAGTGGATTAAGTTCATGAAGGATGGTGTTAATGCCACTCCTCTCCCGACTGTTAA
CACTACTGGTTATATTGACCATGCCGCTTTTCTTGGCACGATTAACCCTGATACCAATAAAATCC
CTAAGCATTTGTTTCAGGGTTATTTGAATATCTATAACAACTATTTTAAAGCGCCGTGGATGCCTG
ACCGTACCGAGGCTAACCCTAATGAGCTTAATCAAGATGATGCTCGTTATGGTTTCCGTTGCTGC
CATCTCAAAAACATTTGGACTGCTCCGCTTCCTCCTGAGACTGAGCTTTCTCGCCAAATGACGAC
TTCTACCACATCTATTGACATTATGGGTCTGCAAGCTGCTTATGCTAATTTGCATACTGACCAAG
AACGTGATTACTTCATGCAGCGTTACCATGATGTTATTTCTTCATTTGGAGGTAAAACCTCTTATG
ACGCTGACAACCGTCCTTTACTTGTCATGCGCTCTAATCTCTGGGCATCTGGCTATGATGTTGAT
GGAACTGACCAAACGTCGTTAGGCCAGTTTTCTGGTCGTGTTCAACAGACCTATAAACATTCTGT
GCCGCGTTTCTTTGTTCCTGAGCATGGCACTATGTTTACTCTTGCGCTTGTTCGTTTTCCGCCTAC
TGCGACTAAAGAGATTCAGTACCTTAACGCTAAAGGTGCTTTGACTTATACCGATATTGCTGGCG
ACCCTGTTTTGTATGGCAACTTGCCGCCGCGTGAAATTTCTATGAAGGATGTTTTCCGTTCTGGT
GATTCGTCTAAGAAGTTTAAGATTGCTGAGGGTCAGTGGTATCGTTATGCGCCTTCGTATGTTTC
TCCTGCTTATCACCTTCTTGAAGGCTTCCCATTCATTCAGGAACCGCCTTCTGGTGATTTGCAAG
AACGCGTACTTATTCGCCACCATGATTATGACCAGTGTTTCCAGTCCGTTCAGTTGTTGCAGTGG
AATAGTCAGGTTAAATTTAATGTGACCGTTTATCGCAATCTGCCGACCACTCGCGATTCAATCAT
GACTTCGTGATAAAAGATTGAGTGTGAGGTTATAACGCCGAAGCGGTAAAAATTTTAATTTTTGC
CGCTGAGGGGTTGACCAAGCGAAGCGCGGTAGGTTTTCTGCTTAGGAGTTTAATCATGTTTCAG
ACTTTTATTTCTCGCCATAATTCAAACTTTTTTTCTGATAAGCTGGTTCTCACTTCTGTTACTCCAG
CTTCTTCGGCACCTGTTTTACAGACACCTAAAGCTACATCGTCAACGTTATATTTTGATAGTTTGA
CGGTTAATGCTGGTAATGGTGGTTTTCTTCATTGCATTCAGATGGATACATCTGTCAACGCCGCT
AATCAGGTTGTTTCTGTTGGTGCTGATATTGCTTTTGATGCCGACCCTAAATTTTTTGCCTGTTTGG
TTCGCTTTGAGTCTTCTTCGGTTCCGACTACCCTCCCGACTGCCTATGATGTTTATCCTTTGGATG
GTCGCCATGATGGTGGTTATTATACCGTCAAGGACTGTGTGACTATTGACGTCCTTCCCCGTACG

CCGGGCAATAATGTTTATGTTGGTTTCATGGTTTGGTCTAACTTTACCGCTACTAAATGCCGCGG
ATTGGTTTCGCTGAATCAGGTTATTAAAGAGATTATTTGTCTCCAGCCACTTAAGTGAGGTGATTT
ATGTTTGGTGCTATTGCTGGCGGTATTGCTTCTGCTCTTGCTGGTGGCGCCATGTCTAAATTGTTT
GGAGGCGGTCAAAAAGCCGCCTCCGGTGGCATTCAAGGTGATGTGCTTGCTACCGATAACAAT
ACTGTAGGCATGGGTGATGCTGGTATTAAATCTGCCATTCAAGGCTCTAATGTTCCTAACCCTGA
TGAGGCCGTCCCTAGTTTTGTTTCTGGTGCTATGGCTAAAGCTGGTAAAGGACTTCTTGAAGGTA
CGTTGCAGGCTGGCACTTCTGCCGTTTCTGATAAGTTGCTTGATTTGGTTGGACTTGGTGGCAAG
TCTGCCGCTGATAAAGGAAAGGATACTCGTGATTATCTTGCTGCTGCATTTCCTGAGCTTAATGC
TTGGGAGCGTGCTGGTGCTGATGCTTCCTCTGCTGGTATGGTTGACGCCGGATTTGAGAATCAA
AAAGAGCTTACTAAAATGCAACTGGACAATCAGAAAGAGATTGCCGAGATGCAAAATGAGACT
CAAAAAGAGATTGCTGGCATTCAGTCGGCGACTTCACGCCAGAATACGAAAGACCAGGTATAT
GCACAAAATGAGATGCTTGCTTATCAACAGAAGGAGTCTACTGCTCGCGTTGCGTCTATTATGG
AAAACACCAATCTTTCCAAGCAACAGCAGGTTTCCGAGATTATGCGCCAAATGCTTACTCAAGC
TCAAACGGCTGGTCAGTATTTTACCAATGACCAAATCAAAGAAATGACTCGCAAGGTTAGTGCT
GAGGTTGACTTAGTTCATCAGCAAACGCAGAATCAGCGGTATGGCTCTTCTCATATTGGCGCTA
CTGCAAAGGATATTTCTAATGTCGTCACTGATGCTGCTTCTGGTGTGGTTGATATTTTTCATGGTA
TTGATAAAGCTGTTGCCGATACTTGGAACAATTTCTGGAAAGACGGTAAAGCTGATGGTATTGG
CTCTAATTTGTCTAGGAAATAACCGTCAGGATTGACACCCTCCCAATTGTATGTTTTCATGCCTCC
AAATCTTGGAGGCTTTTTTATGGTTCGTTCTTATTACCCTTCTGAATGTCACGCTGATTATTTTGAC
TTTGAGCGTATCGAGGCTCTTAAACCTGCTATTGAGGCTTGTGGCATTTCTACTCTTTCTCAATCC
CCAATGCTTGGCTTCCATAAGCAGATGGATAACCGCATCAAGCTCTTGGAAGAGATTCTGTCTTT
TCGTATGCAGGGCGTTGAGTTCGATAATGGTGATATGTATGTTGACGGCCATAAGGCTGCTTCT
GACGTTCGTGATGAGTTTGTATCTGTTACTGAGAAGTTAATGGATGAATTGGCACAATGCTACAA
TGTGCTCCCCCAACTTGATATTAATAACACTATAGACCACCGCCCCGAAGGGGACGAAAAATGG
TTTTTAGAGAACGAGAAGACGGTTACGCAGTTTTGCCGCAAGCTGGCTGCTGAACGCCCTCTTA
AGGATATTCGCGATGAGTATAATTACCCCAAAAAGAAAGGTATTAAGGATGAGTGTTCAAGATT
GCTGGAGGCCTCCACTATGAAATCGCGTAGAGGCTTTGCTATTCAGCGTTTGATGAATGCAATG
CGACAGGCTCATGCTGATGGTTGGTTTATCGTTTTTGACACTCTCACGTTGGCTGACGACCGATT
AGAGGCGTTTTATGATAATCCCAATGCTTTGCGTGACTATTTTCGTGATATTGGTCGTATGGTTCT
TGCTGCCGAGGGTCGCAAGGCTAATGATTCACACGCCGACTGCTATCAGTATTTTTGTGTGCCT
GAGTATGGTACAGCTAATGGCCGTCTTCATTTCCATGCGGTGCACTTTATGCGGACACTTCCTAC
AGGTAGCGTTGACCCTAATTTTGGTCGTCGGGTACGCAATCGCCGCCAGTTAAATAGCTTGCAA
AATACGTGGCCTTATGGTTACAGTATGCCCATCGCAGTTCGCTACACGCAGGACGCTTTTTCACG
TTCTGGTTGGTTGTGGCCTGTTGATGCTAAAGGTGAGCCGCTTAAAGCTACCAGTTATATGGCTG
TTGGTTTCTATGTGGCTAAATACGTTAACAAAAAGTCAGATATGGACCTTGCTGCTAAAGGTCTA
GGAGCTAAAGAATGGAACAACTCACTAAAAACCAAGCTGTCGCTACTTCCCAAGAAGCTGTTCA
GAATCAGAATGAGCCGCAACTTCGGGATGAAAATGCTCACAATGACAAATCTGTCCACGGAGT
GCTTAATCCAACTTACCAAGCTGGGTTACGACGCGACGCCGTTCAACCAGATATTGAAGCAGAA
CGCAAAAAGAGAGATGAGATTGAGGCTGGGAAAAGTTACTGTAGCCGACGTTTTGGCGGCGCA
ACCTGTGACGACAAATCTGCTCAAATTTATGCGCGCTTCGATAAAAATGATTGGCGTATCCAAC
CTGCA
