# Supplementary material for: Study of Endogenous Viruses in the Strawberry Plants
Source: Viruses. 2024 Aug 16;16(8):1306. doi: 10.3390/v16081306 (PMC11359110; doi:10.3390/v16081306)
Supplement: Supplementary file 1 [file viruses-16-01306-s001.zip › Supplementary table s1.pdf]

Supplementary Table s1 The plant genomes of *Fragaria* and its related genera used in this study

number	Species	Accession No.	Size(MB)	Assembly level
Fragaria				
01	*Fragaria × ananassa*	GCA_019022445.1	805.7	Chromosome
02	*Fragaria orientalis*	GCA_000517285.1	214.2	Scaffold
03	*Fragaria iinumae*	GCA_009720345.1	240.6	Chromosome
04	*Fragaria nilgerrensis*	GCA_010134655.1	270.3	Chromosome
05	*Fragaria vesca*	GCF_000184155.1	214.2	Chromosome
06	*Fragaria nubicola*	GDR (rosaceae.org)	238.0	Chromosome
07	*Fragaria nipponica*	GDR (rosaceae.org)	275.0	Chromosome
08	*Fragaria daltoniana*	GDR (rosaceae.org)	288.97	Chromosome
09	*Fragaria mandshurica*	GDR (rosaceae.org)	239.83	Chromosome
10	*Fragaria viridis*	GDR (rosaceae.org)	223.08	Chromosome
11	*Fragaria chiloensis*	GDR (rosaceae.org)	212.0	Chromosome
12	*Fragaria pentaphylla*	GDR (rosaceae.org)	279.04	Chromosome
13	*Fragaria. virginiana*	GDR (rosaceae.org)	717.0	Chromosome
14	*Fragaria moupinensis*	GDR (rosaceae.org)	868.0	Chromosome
Potentilla				
1	*Potentilla anserina*	GCA_933775445.1	228.0	Chromosome
2	*Potentilla sterilis*	GCA_963682095.1	719.0	Chromosome
Rubus				
1	*Rubus argutus*	GDR (rosaceae.org)	289.0	Chromosome
2	*Rubus chingii*	GDR (rosaceae.org)	228.0	Chromosome
3	*Rubus Idaeus*	GDR (rosaceae.org)	288.0	Chromosome
4	*Rubus occidentalis*	GDR (rosaceae.org)	280.0	Chromosome
