# Supplementary material for: Study of Endogenous Viruses in the Strawberry Plants
Source: Viruses. 2024 Aug 16;16(8):1306. doi: 10.3390/v16081306 (PMC11359110; doi:10.3390/v16081306)
Supplement: Supplementary file 1 [file viruses-16-01306-s001.zip › Supplementary Table s2.pdf]

Supplementary Table s1 The probe sequences used for homologous search

Virus	Classification (Family/Genus)	Region	Origin	Accession No.
Cauliflower mosaic virus	*Caulimoviridae Caulimovirus*	RT	NCBI	NP_056728.1
Rose yellow vein virus	*Caulimoviridae Rosadnavirus*	RT	NCBI	YP_007761644.1
Petunia vein clearing virus	*Caulimoviridae Petuvirus*	RT	NCBI	Q6XKE6
Dioscorea nummularia-associated virus	*Caulimoviridae Dioscovirus*	RT	NCBI	YP_009553219
Grapevine badnavirus 1	*Caulimoviridae Badnavirus*	RT	https://www.uniprot.org/	A0A2H4N978
Blueberry fruit drop associated virus	*Caulimoviridae Vaccinivirus*	RT	https://www.uniprot.org/	A0A0S2A4A7
Blueberry red ringspot virus	*Caulimoviridae Soymovirus*	RT	https://www.uniprot.org/	G5CKJ8
Rice tungro bacilliform virus	*Caulimoviridae Tungrovirus*	RT	https://www.uniprot.org/	C4QUK2
Rudbeckia flower distortion virus	*Caulimoviridae Ruflodivirus*	RT	https://www.uniprot.org/	B8Y871
Sweet potato collusive virus	*Caulimoviridae Cavemovirus*	RT	https://www.uniprot.org/	E5KBV1
Tobacco vein clearing virus	*Caulimoviridae Solendovirus*	RT	https://www.uniprot.org/	Q9QD04
retrovirus element Athila4	*Metaviridae*	RT	NCBI	AF378081.1
Alfalfa leaf curl virus	*Geminiviridae Capulavirus*	Rep	https://www.uniprot.org/	A0A166V1S2
Apple geminivirus	*Geminiviridae Maldovirus*	Rep	https://www.uniprot.org/	A0A858M4E9
Bean golden yellow mosaic virus	*Geminiviridae Begomovirus*	Rep	https://www.uniprot.org/	P0CK40
Beet curly top Iran virus	*Geminiviridae Becurtovirus*	Rep	https://www.uniprot.org/	B1P3D6
Beet curly top virus	*Geminiviridae Curtovirus*	Rep	https://www.uniprot.org/	P14991
Eragrostis curvula streak virus	*Geminiviridae Eragrovirus*	Rep	https://www.uniprot.org/	C3UV60
Grapevine redleaf-associated virus	*Geminiviridae Grablovirus*	Rep	https://www.uniprot.org/	T2B2N5
Maize streak virus	*Geminiviridae Mastrevirus*	Rep	https://www.uniprot.org/	P14988
Opuntia virus 1	*Geminiviridae Opunvirus*	Rep	https://www.uniprot.org/	A0A6C0M7U4
Passion fruit chlorotic mottle virus	*Geminiviridae Citlodavirus*	Rep	https://www.uniprot.org/	A0A2R4Q8U9
Tomato apical leaf curl virus	*Geminiviridae Topilevirus*	Rep	https://www.uniprot.org/	A0A3S9JKY8
Tomato pseudo-curly top virus	*Geminiviridae Topocuvirus*	Rep	https://www.uniprot.org/	Q88888
Turnip leaf roll virus	*Geminiviridae Turncurtovirus*	Rep	https://www.uniprot.org/	A0A0S3JNW4
Strawberry crinkle virus (ssRNA(-))	*Rhabdoviridae Cytorhabdovirus*	RDRP	NCBI	AAP03645.2
Strawberry mottle virus (ssRNA(+))	*Secoviridae Sadwavirus*	RDRP	NCBI	QPZ44443.1
Fragaria chiloensis cryptic virus (dsRNA)	*Partitiviridae*	RDRP	NCBI	QZN83648.1
