# Supplementary material for: Study of Endogenous Viruses in the Strawberry Plants
Source: Viruses. 2024 Aug 16;16(8):1306. doi: 10.3390/v16081306 (PMC11359110; doi:10.3390/v16081306)
Supplement: Supplementary file 1 [file viruses-16-01306-s001.zip › Supplementary Table s3.pdf]

Supplementary Table s1 Overview of the search for homologs of viral replicases in the genomes of the *Fragaria* and its closely related plant genera.

category	Caulimovirida (dsDNA)	Geminiviridae (ssDNA)	Metaviridae (LTR retrotransposon)	*Fragaria chiloensis cryptic virus* (dsRNA)	*Strawberry crinkle virus* (ssRNA(-)	*Strawberry mottle virus* (ssRNA(+))
Fragaria	√		√			
Potentilla	√		√	√		
Rubus	√		√			

Note: Sequences in the strawberry genomes with at least 50% amino acid similarity and a minimum length of 150 amino acids to the virus replicase were considered homologous to the virus replicase. "√" indicates the presence of homologous sequences to the virus replicase in the corresponding plant group's genomes, while a blank space denotes the absence of relevant sequences.
