# Supplementary material for: Study of Endogenous Viruses in the Strawberry Plants
Source: Viruses. 2024 Aug 16;16(8):1306. doi: 10.3390/v16081306 (PMC11359110; doi:10.3390/v16081306)
Supplement: Supplementary file 1 [file viruses-16-01306-s001.zip › Supplementary Table s4.pdf]

Supplementary Table s1 Statistical results of plant pararetrovirus-like sequences and effective sequences identified by homologs search in the strawberry genomes

Strawberry species	Pararetrovirus-like sequences		EPRVs	
	Quantity of sequences	Cluster	Effective sequences	Cluster
Fragaria ananassa	70	11	33	5
Fragaria orientalis	46	7	24	5
Fragaria virginiana	78	11	33	5
Fragaria iinumae	53	7	26	4
Fragaria nilgerrensis	97	12	34	4
Fragaria mandschurica	56	9	25	5
Fragaria viridis	57	14	27	6
Fragaria daltoniana	50	9	23	4
Fragaria nipponica	176	16	81	8
Fragaria pentaphylla	157	13	70	8
Fragaria moupinensis	551	15	276	7
Fragaria nubicola	126	12	70	6
Fragaria vesca	37	12	16	7
Fragaria chiloensis	94	11	44	5
