# Supplementary material for: Study of Endogenous Viruses in the Strawberry Plants
Source: Viruses. 2024 Aug 16;16(8):1306. doi: 10.3390/v16081306 (PMC11359110; doi:10.3390/v16081306)
Supplement: Supplementary file 1 [file Supplementary Figure s1.pdf › Supplementary Figure s1.pdf]

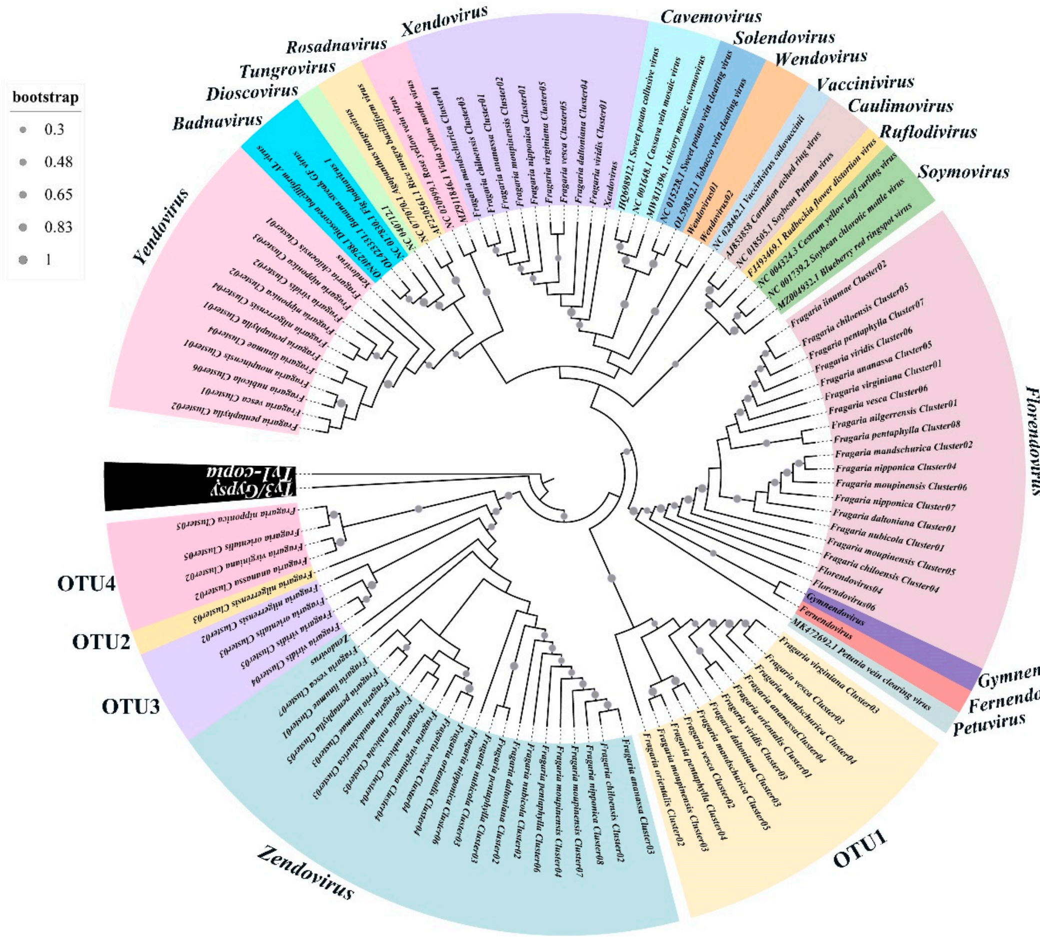

supplementary Figure s1: the rooted tree of strawberry pararetroviral RT homologs within the *Caulimoviridae* family, got from homology search.
